# Supplementary material for: Exploration of Challenges and Opportunities for Good Pharmacy Practices in Bangladesh: A Qualitative Study
Source: Pharmacy (Basel). 2025 Feb 13;13(1):26. doi: 10.3390/pharmacy13010026 (PMC11859208; doi:10.3390/pharmacy13010026)
Supplement: Supplementary file 1 [file pharmacy-13-00026-s001.zip › pharmacy-3338719-supplementary.pdf]

pharmacy

MDPI

Supplementary Materials

Annex S1: Interview topic guide of Grade A and Grade B pharmacists

Attitude and opinion	• How you been recruited or employed in the model pharmacy?<br>• What is your role in the model pharmacy?<br>• How do you manage the pharmacy?<br>• How many staffs are currently working under your supervision?<br>• What are your feelings about your job as Pharmacist?<br>• What is your opinion about the sustainability of Grade A pharmacist in the model pharmacy?
Motivation status (job status (social), recognition, existing facilities, reason for discontinuation)	• How do you describe your motivation status as Grade A pharmacist in the model pharmacy?<br>• Are you satisfied or dissatisfied about your job (including social and economic point of view)? Why?<br>• What are the current facilities (salaries and other benefits) of your job?<br>• Why did you leave the job from the model pharmacy (if anyone left the job)
Barriers, challenges and opportunities	• What are the common barriers and challenges of a Grade A pharmacist in the model pharmacy?<br>• How do you (did you) manage the challenges (please describe)<br>• How can overcome the barriers and challenges in operating model pharmacies<br>• What are the potential opportunities for Grade A pharmacist in the model pharmacy for improving dispensing facilities
Recommendations about the retention and sustainability	• What are your recommendations for the sustainability of Grade A pharmacist in the model pharmacy (please describe)?<br>• Are there any options to manage model pharmacies without Grade A pharmacist?<br>• Can Grade B pharmacist manage the model pharmacy? What are your suggestions for placing Grade B pharmacist as pharmacy-in-charge at model pharmacy?
Perceptions and understandings about good pharmacy practice	• What is your perception about the good pharmacy practice?<br>• What are the opportunities of improving pharmacy practice?

Annex S2: Interview topic guide of stakeholders

Model Pharmacy and Model Medicine shop	• What do you know about the model pharmacy and model medicine shops in Bangladesh? Please describe.<br>• Who are the key persons in the model pharmacy and model medicine shops?<br>• What are their roles in model pharmacies and model medicine shops?<br>• What are the reasons for discontinuation of Grade A pharmacist in the model pharmacies?
Opinion about of Grade B Pharmacist placement as in-charge	• Who are the potential options in the model pharmacies instead of Grade A pharmacist?<br>• What is your opinion about the pharmacy-in-charge of Grade B pharmacist in the model pharmacy?<br>• Are they potential or suitable? How and why?<br>• Is there any problem becoming pharmacy-in-charge of Grade B pharmacist in the model pharmacy? What are the problems?
Opinion and recommendation for Grade B Pharmacist curriculum	• Do you think Grade B pharmacist current curriculum is fit for standard pharmacy practice in Bangladesh? If yes/no, Why?<br>• Is the current curriculum sufficient for better pharmacy practice in Bangladesh? If yes how?<br>• If no, how the curriculum can be upgraded for better pharmacy practice in Bangladesh?<br>• What are your recommendations for upgrading Grade B pharmacy curriculum? How it can be done? Please describe.
Recommendations about the sustainability of Grade A pharmacist in the model pharmacy	• What are your recommendations for sustainability of Grade A pharmacist in the model pharmacy?<br>• Please ask about-recognition, facilities, designation, status, opportunities (learning and capacity building), working hours, etc.
Recommendations for improving good pharmacy practice	• What are the challenges for good pharmacy practice in Bangladesh?<br>• What are your recommendations for improving pharmacy practice in Bangladesh?
